# National Racial and Sex Disparities in Primary Total Hip and Knee Arthroplasty Utilization: A Nationwide Analysis, 2016-2021

**DOI:** 10.7759/cureus.113044

**Published:** 2026-07-20

**Authors:** George Thomas, Mariah Norling, Paul Lender, Paul Hoogervorst

**Affiliations:** 1 Department of Orthopaedic Surgery, University of Minnesota, Minneapolis, USA

**Keywords:** health disparities, hip arthroplasty, knee arthroplasty, race, sex, total joint arthroplasty

## Abstract

Total joint arthroplasty (TJA) is one of the most commonly performed elective surgical procedures in the United States. Prior studies up to 2015 have found racial disparities in utilization to exist despite a higher osteoarthritis burden among Black patients. The intersection of race and sex disparities in TJA utilization is poorly understood.

Using the 2016-2021 Healthcare Cost and Utilization Project Nationwide Inpatient Sample (HCUP-NIS), primary elective total hip arthroplasty (THA) and total knee arthroplasty (TKA) procedures for degenerative joint disease were identified. Age-adjusted Black and White utilization rates, using U.S. Census data, were stratified by sex. Patient, hospital, and outcome variables were compared, and the temporal inpatient trends were analyzed using linear regression models adjusted for the COVID-19 pandemic.

Of the 4,067,950 patients, Black patients were younger, more likely to be in the lowest income quartile, have Medicaid insurance, and have undergone surgery at urban teaching hospitals than White patients (all p < 0.001). They had longer hospital stays and higher morbidity (p < 0.001). In 2016, THA utilization was 9.8 per 10,000 for White individuals compared with 4.3 for Black individuals; this disparity increased over the study period (p = 0.01). White women had the highest utilization rates, and Black men had the lowest. Disparities between White and Black women in THA utilization were found to increase (p = 0.03), whereas disparities between White and Black men remained consistent. For TKA, utilization was 19.1 per 10,000 for White individuals compared with 9.4 for Black individuals in 2016. White-Black, White-Black men, and White-Black women disparities in utilization remained consistent over the study period.

From 2016 to 2021, racial disparities in inpatient TKA persisted, while disparities in inpatient THA utilization worsened, with pronounced differences found at the intersection of race and sex. These findings highlight the need for targeted, equity-focused interventions to improve access to TJA.

## Introduction

Over one million elective total joint arthroplasty (TJA) procedures are performed annually in the United States [1]. With an aging population and increased demand, the volumes of annual elective total hip arthroplasties (THAs) and total knee arthroplasties (TKAs) are expected to grow by 176% and 139%, respectively, by 2040 [2]. Studies have shown that Black patients have an increased risk of symptomatic and radiographic hip and knee osteoarthritis compared to White patients [3,4]. However, persistent White-Black disparities in elective TJA utilization rates and outcomes have been documented up until 2015 [5-8]. Following this, multiple interventions arose in 2010 to address these disparities [9].

Prior studies indicate that women are less likely than men to receive a referral for TJA evaluation in the setting of osteoarthritis. They are also less likely to be recommended for TJA by their surgeon [10]. Population-based studies in both Canada and Europe have found a general decrease in TJA utilization among women [11,12]. The intersection of race and sex in TJA utilization rates has not been well characterized. Understanding how these factors intersect is essential for identifying which patients are least likely to reach surgical consultation and for improving equity in both referral and treatment pathways [13].

In this study, we used the 2016-2021 Healthcare Cost and Utilization Project Nationwide Inpatient Sample (HCUP-NIS) data to examine race and sex disparities in age-adjusted THA and TKA utilization rates. Our primary aim was to examine White versus Black, White men versus Black men, and White women versus Black women THA/TKA utilization rates. Secondary outcomes included studying these disparities in patient characteristics, hospital characteristics, and postoperative outcomes.

## Materials and methods

The HCUP-NIS, sponsored by the Department of Health and Human Services’ (DHHS) Agency for Healthcare Research and Quality, samples data from all-payer hospital admissions in 48 states, representing 97% of the US population [14]. Hospitals transitioned from reporting diagnosis and procedure codes with the ICD-9 system to the ICD-10 system in October 2015. The first complete annual HCUP-NIS dataset reporting ICD-10 codes was available in 2016 [15]. The datasets from 2016 to 2021 were queried for all patients who underwent a primary elective THA or TKA for osteoarthritis using relevant ICD-10 codes. TJAs for revision, malignancy, trauma, and infection were excluded (Table 1). 

**Table 1:** HCUP ICD-10 inclusion and exclusion codes for primary TKA and THA. HCUP: Healthcare Cost and Utilization Project; TKA: Total Knee Arthroplasty; THA: Total Hip Arthroplasty

Purpose	Code Type	ICD-10 Codes
Inclusion - Primary Total Hip Arthroplasty	ICD-10-PCS	0SR90, 0SRB0
Inclusion - Primary Total Knee Arthroplasty	ICD-10-PCS	0SRC0, 0SRT0, 0SRV0, 0SRD0, 0SRU0, 0SRW0
Exclusion - Infection	ICD-10-CM	A0472, A401, A408, A4101, A4150, A4151, A4159, A4181, A4189, A419, D16, D21, D492, D649, L02415, L03116, M00051, M00052, M00851, M00852, M00862, M009, M12, M4628, M60003, M80, M84, M868X5, M87, M96, T8140XA, T8141XA, T8142XA, T8149XA
Exclusion - Trauma/Fracture	ICD-10-CM	M9701XA, M9702XA, M9712XA, S32, S72, S73004A, S79, S82041C, S82131A, S82141A, S82142A, S82142S, S82201A
Exclusion - Neoplasm	ICD-10-CM	C40, C419, C47, C49, C7951, C9000
Exclusion - Revision Arthroplasty	ICD-10-PCS	0SP90JZ, 0SPB0JZ, 0SPC0JZ, 0SPD0JZ, 0SW90JZ, 0SWB0JZ, 0SWC0JZ, 0SWD0JZ
Exclusion - Other Arthroplasty Complications	ICD-10-CM	Q65, T8131XA, T8132XA, T8133XA, T8189XA, T84010A-T84093A, T84114A-T84195A, T84218A, T84298A, T84328A, T84418A, T84428A, T84498A, T8450XA-T8459XD, T84620A, T84621A, T84622A, T847XXA, T8482XA-T8489XD, T849XXA, T85628A, T85848A, T859XXA

**Figure 1:**
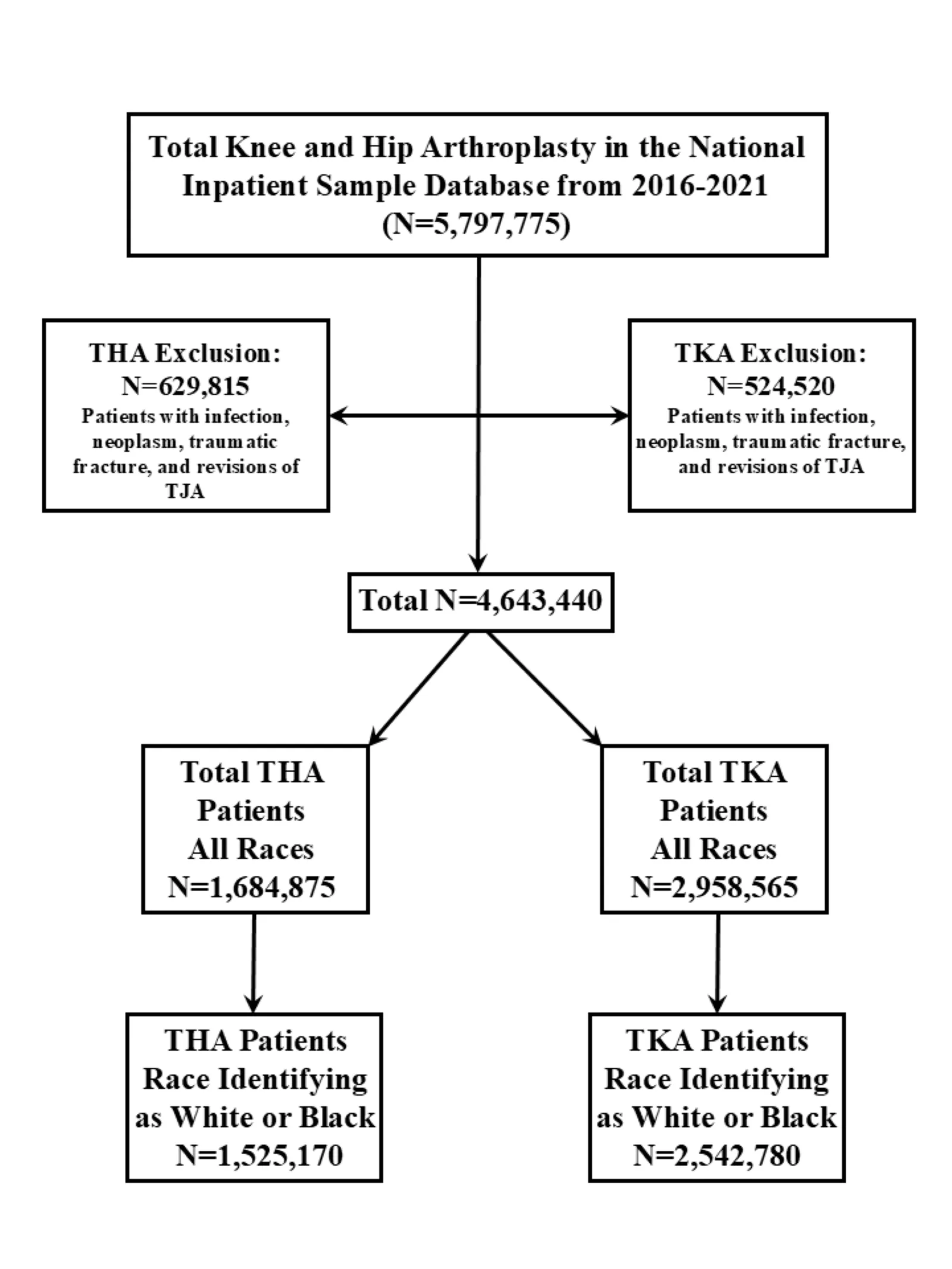
Criteria for excluding primary THA and primary TKA within this study. TKA: Total Knee Arthroplasty; THA: Total Hip Arthroplasty

Patients who did not report White or Black race were excluded from further analysis (12.4%). A total of 4,067,950 patients were included for analysis (Figure 1). This study was deemed exempt from IRB approval because it incorporates de-identified, publicly available HCUP-NIS data without direct human subject involvement.

Primary outcomes were the annual age-adjusted THA and TKA utilization rates for White, Black, White men, Black men, White women, and Black women. For each study year (2016-2021), crude utilization rates were calculated by dividing the annual number of THA or TKA procedures within each age-, race-, and sex-specific cohort by the corresponding annual U.S. Census population estimate obtained from the American Community Survey and were expressed per 10,000 population [16]. Age-specific crude rates were then directly standardized to the 2000 U.S. standard population using the direct standardization method. Secondary outcomes included the preoperative demographics, hospital characteristics, and postoperative outcomes shown in Table 2. Postoperative morbidity was reported using a severity of illness score from 0 to 4, with 4 indicating extreme loss of function.

**Table 2:** Patient demographics, hospital characteristics, and postoperative outcomes by race for primary TKA and THA. THA: Total Hip Arthroplasty; TKA: Total Knee Arthroplasty

			Primary THA				Primary TKA			
			White	Black	Test statistic	p-value	White	Black	Test statistic	p-value
No. of patients			1,404,915	120,255	-	-	2,315,555	227,225	-	-
Age, Yr	Median		67	62	t(1,525,168) = 235.0	<0.001	67	63	t(2,542,778) = 189.0	<0.001
	≤65 yr, No. (%)		550,230 (39.2)	69,755 (58.0)	χ²(2) = 17,000	<0.001	862,485 (37.2)	124,190 (54.7)	χ²(2) = 27,000	<0.001
	65-79 yr, No. (%)		705,055 (50.2)	44,730 (37.2)			1,238,165 (53.5)	91,940 (40.5)		
	≥80 yr, No. (%)		149,630 (10.7)	5,770 (4.8)			214,905 (9.3)	11,095 (4.9)		
Sex	Men, No. (%)		623,330 (44.4)	53,580 (44.6)	χ²(1) = 1.52	0.22	929,485 (40.1)	65,530 (28.8)	χ²(1) = 11,000	<0.001
	Women, No. (%)		781,410 (55.6)	66,670 (55.4)			1,385,695 (59.9)	161,620 (71.2)		
Income	1st quartile, No. (%)		230,035 (16.6)	52,605 (44.4)	χ²(3) = 58,000	<0.001	443,040 (19.4)	102,130 (45.7)	χ²(3) = 86,000	<0.001
	2nd quartile, No. (%)		351,810 (25.4)	25,995 (22.0)			621,865 (27.2)	50,815 (22.7)		
	3rd quartile, No. (%)		388,955 (28.1)	22,420 (18.9)			637,425 (27.9)	41,500 (18.6)		
	4th quartile, No. (%)		415,400 (30.0)	17,330 (14.6)			582,810 (25.5)	29,085 (13.0)		
Insurance Status	Medicare, No. (%)		805,725 (57.4)	53,895 (44.9)	χ²(3) = 25,000	<0.001	1,358,225 (58.7)	107,580 (47.4)	χ²(3) = 36,000	<0.001
	Medicaid, No. (%)		47,315 (3.4)	14,280 (11.9)			69,390 (3.0)	22,320 (9.8)		
	Private, No. (%)		510,470 (36.4)	45,440 (37.9)			806,310 (34.9)	82,705 (36.4)		
	Other, No. (%)		39,470 (2.8)	6,390 (5.3)			78,770 (3.4)	14,330 (6.3)		
Hospital Location/Type	Rural, No. (%)		121,470 (8.6)	4,955 (4.1)	χ²(2) = 6,500	<0.001	263,985 (11.4)	12,635 (5.6)	χ²(2) = 16,000	<0.001
	Urban non-teaching, No. (%)		354,015 (25.2)	22,675 (18.9)			637,590 (27.5)	46,105 (20.3)		
	Urban teaching, No. (%)		929,430 (66.2)	92,625 (77.0)			1,413,980 (61.1)	168,485 (74.1)		
Hospital Size	Small, No. (%)		457,655 (32.6)	34,580 (28.8)	χ²(2) = 790	<0.001	768,285 (33.2)	64,255 (28.3)	χ²(2) = 2,300	<0.001
	Medium, No. (%)		384,025 (27.3)	33,695 (28.0)			645,215 (27.9)	67,960 (29.9)		
	Large, No. (%)		563,235 (40.1)	51,980 (43.2)			902,055 (39.0)	95,010 (41.8)		
Mean Severity (0-4), SD			1.5 (0.6)	1.6 (0.6)	t(1,525,168) = -55.5	<0.001	1.5 (0.6)	1.5 (0.6)	t(2,542,778) = -60.7	<0.001
Mean LOS (days), SD			1.9 (1.7)	2.3 (2.0)	t(1,525,168) = -67.3	<0.001	2.1 (1.6)	2.5 (2.0)	t(2,542,778) = -92.5	<0.001

Statistical analysis

THA and TKA patients were studied separately. The numbers used were weighted counts from statistical software survey commands. Chi-square and rank sum statistics were used to identify White-Black, White men-Black men, and White women-Black women disparities in preoperative demographics, hospital characteristics, and postoperative outcomes. For each variable, the sum of events over all six years was used for analysis. Age-adjusted utilization rates were charted for each cohort across the study period per 10,000 people. Linear regression analysis, adjusted for the presence of the COVID-19 pandemic in 2020 and 2021, was used to identify trends in the age-adjusted rates and disparities. To adjust for COVID-19, a binary indicator variable was included in the regression models for the years 2016-2019 versus 2020-2021. This indicator was included in the regression model along with calendar year and a year with COVID-19 interaction term. Statistical significance was defined as p < 0.05. Stata 19.5 (StataCorp LLC, College Station, TX, USA) was used for statistical analysis [17].

## Results

THA disparities in patient characteristics, hospital characteristics, and postoperative outcomes

Of 1,525,170 THA patients, 92.1% were White (Table 2). Of the White patients, 55.6% were women versus 55.4% of Black patients (χ²(1) = 1.52, p = 0.22). Black patients were significantly more likely to be younger than 65 years (58.0% versus 39.2% of White patients; χ²(2) = 17,000, p < 0.001), to be in the lowest median household income quartile (44.4% versus 16.6% of White patients; χ²(3) = 58,000, p < 0.001), and to have Medicaid (11.9% versus 3.4% of White patients; χ²(3) = 25,000, p < 0.001). Among Black patients, 77.0% received care at urban teaching hospitals versus 66.2% of White patients (χ²(2) = 6,500, p < 0.001), although Black patients were more likely to receive care at larger hospitals (43.2% versus 40.1% of White patients; χ²(2) = 790, p < 0.001). Black patients had significantly longer hospital stays (t(1,525,168) = -67.3, p < 0.001) and higher postoperative morbidity (t(1,525,168) = -55.5, p < 0.001). Similar disparities were seen in the stratified analysis by sex for Black men and Black women (Table 3).

**Table 3:** Patient demographics, hospital characteristics, postoperative outcomes by race and sex for primary THA. THA: Total Hip Arthroplasty

		Primary THA							
		Men				Women			
		White	Black	Test statistic	p-value	White	Black	Test statistic	p-value
No. of patients		623,330	53,580	-		781,410	66,670	-	
Age, Yr	Median	66	60	t(676,908) = 138.0	<0.001	68	64	t(848,078) = 95.6	<0.001
	≤65 yr, No. (%)	277,755 (44.6)	35,395 (66.1)	χ²(2) = 9,600	<0.001	272,425 (34.9)	34,360 (51.5)	χ²(2) = 8,000	<0.001
	65-79 yr, No. (%)	291,480 (46.8)	16,545 (30.9)			413,465 (52.9)	28,185 (42.3)		
	≥80 yr, No. (%)	54,095 (8.7)	1,640 (3.1)			95,520 (12.2)	4,125 (6.2)		
Income	1st quartile, No. (%)	98,900 (16.1)	22,700 (43.1)	χ²(3) = 25,000	<0.001	131,125 (17.0)	29,905 (45.6)	χ²(3) = 33,000	<0.001
	2nd quartile, No. (%)	154,500 (25.1)	11,720 (22.2)			197,255 (25.6)	14,270 (21.7)		
	3rd quartile, No. (%)	172,335 (28.0)	10,285 (19.5)			216,575 (28.1)	12,135 (18.5)		
	4th quartile, No. (%)	188,870 (30.7)	8,005 (15.2)			226,470 (29.4)	9,325 (14.2)		
Insurance Status	Medicare, No. (%)	317,630 (51.0)	19,500 (36.5)	χ²(3) = 16,000	<0.001	487,975 (62.5)	34,390 (51.7)	χ²(3) = 10,000	<0.001
	Medicaid, No. (%)	21,630 (3.5)	7,330 (13.7)			25,685 (3.3)	6,950 (10.4)		
	Private, No. (%)	259,210 (41.7)	22,640 (42.4)			251,205 (32.2)	22,800 (34.2)		
	Other, No. (%)	23,825 (3.8)	3,960 (7.4)			15,645 (2.0)	2,430 (3.7)		
Hospital Location/Type	Rural, No. (%)	54,160 (8.7)	2,210 (4.1)	χ²(2) = 2,600	<0.001	67,310 (8.6)	2,745 (4.1)	χ²(2) = 3,900	<0.001
	Urban non-teaching, No. (%)	156,930 (25.2)	10,460 (19.5)			197,050 (25.2)	12,215 (18.3)		
	Urban teaching, No. (%)	412,240 (66.1)	40,910 (76.4)			517,050 (66.2)	51,710 (77.6)		
Hospital Size	Small, No. (%)	203,720 (32.7)	15,745 (29.4)	χ²(2) = 290	<0.001	253,845 (32.5)	18,835 (28.3)	χ²(2) = 517	<0.001
	Medium, No. (%)	169,765 (27.2)	14,650 (27.3)			214,230 (27.4)	19,045 (28.6)		
	Large, No. (%)	249,845 (40.1)	23,185 (43.3)			313,335 (40.1)	28,790 (43.2)		
Mean Severity (0-4), SD		1.5 (0.6)	1.5 (0.6)	t(676,908) = -22.5	<0.001	1.5 (0.6)	1.6 (0.6)	t(848,078) = -58.0	<0.001
Mean LOS (days), SD		1.7 (1.8)	2.1 (2.0)	t(676,908) = -44.8	<0.001	2.0 (1.6)	2.4 (2.0)	t(848,078) = -54.8	<0.001

TKA disparities in patient characteristics, hospital characteristics, and postoperative outcomes

Of 2,542,780 TKA patients, 91.1% of patients were White (Table 2). Women made up 59.9% of White patients compared with 71.2% of Black patients (χ²(1) = 11,000, p < 0.001). Significant disparities were observed between White and Black patients in age distribution (χ²(2) = 27,000, p < 0.001), income quartile (χ²(3) = 86,000, p < 0.001), insurance status (χ²(3) = 36,000, p < 0.001), hospital characteristics, and postoperative outcomes (all p < 0.001) (Table 4).

**Table 4:** Patient demographics, hospital characteristics, postoperative outcomes by race and sex for primary TKA. TKA: Total Knee Arthroplasty

		Primary TKA							
		Men				Women			
		White	Black	Test statistic	p-value	White	Black	Test statistic	p-value
No. of patients		929,485	65,530	-		1,385,695	161,620	-	
Age, Yr	Median	67	62	t(995,013) = 139.0	<0.001	68	64	t(1,546,313) = 158.0	<0.001
	≤65 yr, No. (%)	358,995 (38.6)	38,005 (58.0)	χ²(2) = 9,800	<0.001	503,335 (36.3)	86,150 (53.3)	χ²(2) = 18,000	<0.001
	65-79 yr, No. (%)	488,990 (52.6)	24,735 (37.7)			748,985 (54.1)	67,165 (41.6)		
	≥80 yr, No. (%)	81,500 (8.8)	2,790 (4.3)			133,375 (9.6)	8,305 (5.1)		
Income	1st quartile, No. (%)	171,500 (18.7)	27,275 (42.4)	χ²(3) = 22,000	<0.001	271,490 (19.8)	74,815 (47.0)	χ²(3) = 62,000	<0.001
	2nd quartile, No. (%)	246,260 (26.9)	15,080 (23.4)			375,520 (27.4)	35,720 (22.5)		
	3rd quartile, No. (%)	257,310 (28.1)	12,550 (19.5)			380,005 (27.8)	28,935 (18.2)		
	4th quartile, No. (%)	241,005 (26.3)	9,490 (14.7)			341,680 (25.0)	19,590 (12.3)		
Insurance Status	Medicare, No. (%)	506,175 (54.5)	26,350 (40.3)	χ²(3) = 16,000	<0.001	851,855 (61.5)	81,200 (50.3)	χ²(3) = 22,000	<0.001
	Medicaid, No. (%)	22,685 (2.4)	5,955 (9.1)			46,695 (3.4)	16,355 (10.1)		
	Private, No. (%)	352,475 (38.0)	26,020 (39.8)			453,670 (32.8)	56,655 (35.1)		
	Other, No. (%)	46,620 (5.0)	7,070 (10.8)			32,145 (2.3)	7,255 (4.5)		
Hospital Location/Type	Rural, No. (%)	105,470 (11.3)	3,710 (5.7)	χ²(2) = 4,100	<0.001	158,485 (11.4)	8,915 (5.5)	χ²(2) = 12,000	<0.001
	Urban non-teaching, No. (%)	256,985 (27.6)	13,975 (21.3)			380,505 (27.5)	32,115 (19.9)		
	Urban teaching, No. (%)	567,030 (61.0)	47,845 (73.0)			846,705 (61.1)	120,590 (74.6)		
Hospital Size	Small, No. (%)	310,760 (33.4)	19,520 (29.8)	χ²(2) = 371	<0.001	457,385 (33.0)	44,715 (27.7)	χ²(2) = 1,900	<0.001
	Medium, No. (%)	257,885 (27.7)	18,965 (28.9)			387,225 (27.9)	48,980 (30.3)		
	Large, No. (%)	360,840 (38.8)	27,045 (41.3)			541,085 (39.0)	67,925 (42.0)		
Mean Severity (0-4), SD		1.4 (0.6)	1.5 (0.6)	t(995,013) = -24.2	<0.001	1.5 (0.6)	1.6 (0.6)	t(1,546,313) = -50.0	<0.001

THA disparities in utilization rates

**Figure 2:**
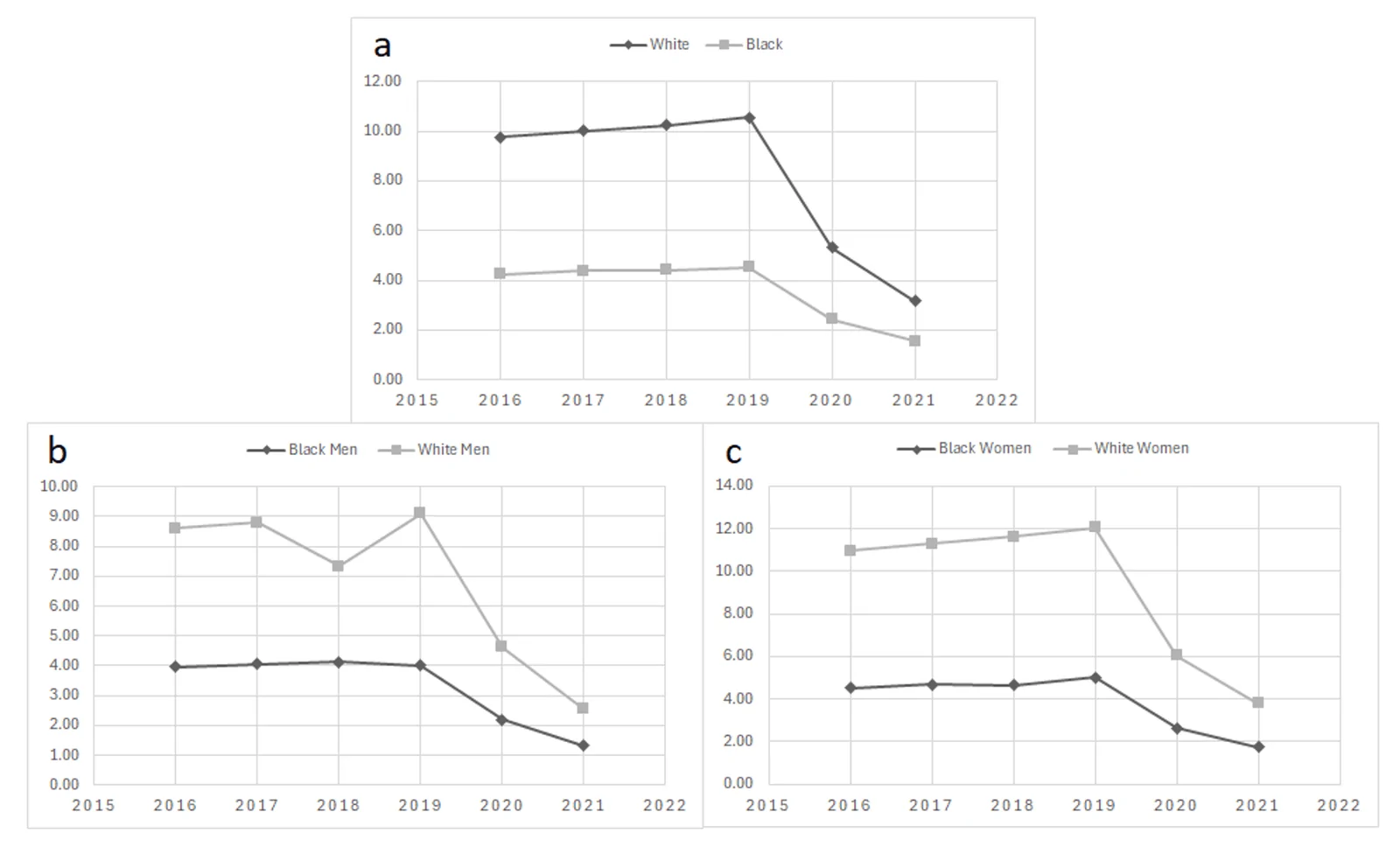
Age-adjusted THA utilization disparities per 10,000 people by race and sex from 2016 to 2021. Panel (a): Trends for all White versus Black patients. Disparity slope = 0.2, 95% CI (0.1, 0.2), p = 0.01*. Panel (b): Trends for all White men versus Black men. Disparity slope = -0.02, 95% CI (-1.9, 1.9), p = 1.0. Panel (c): Trends for all White women versus Black women. Disparity slope = 0.2, 95% CI (0.1, 0.4), p = 0.03*. *Statistically significant changes in disparity.

Age-adjusted THA utilization rates were 129.8% higher in White patients than in Black patients in 2016 (9.8 per 10,000 White patients, 4.3 per 10,000 Black patients). Despite rates increasing in both groups to 2019 (10.6 per 10,000 White patients, 4.5 per 10,000 Black patients), the White-Black disparity significantly worsened over the study period (p = 0.01) (Figure 2). In 2016, rates were 117.0% higher in White men than in Black men (8.6 per 10,000 White men, 4.0 per 10,000 Black men) and 142.1% higher in White women than in Black women (11.0 per 10,000 White women, 4.5 per 10,000 Black women). Age-adjusted utilization rates increased across all cohorts except for Black men, whose rates remained steady and were consistently the lowest. White women consistently had the highest utilization rates. The White women-Black women disparity significantly worsened (p = 0.03). No significant change in the White men-Black men disparity (p = 0.98) was observed over the study period (Figure 2). 

TKA disparities in utilization rates

**Figure 3:**
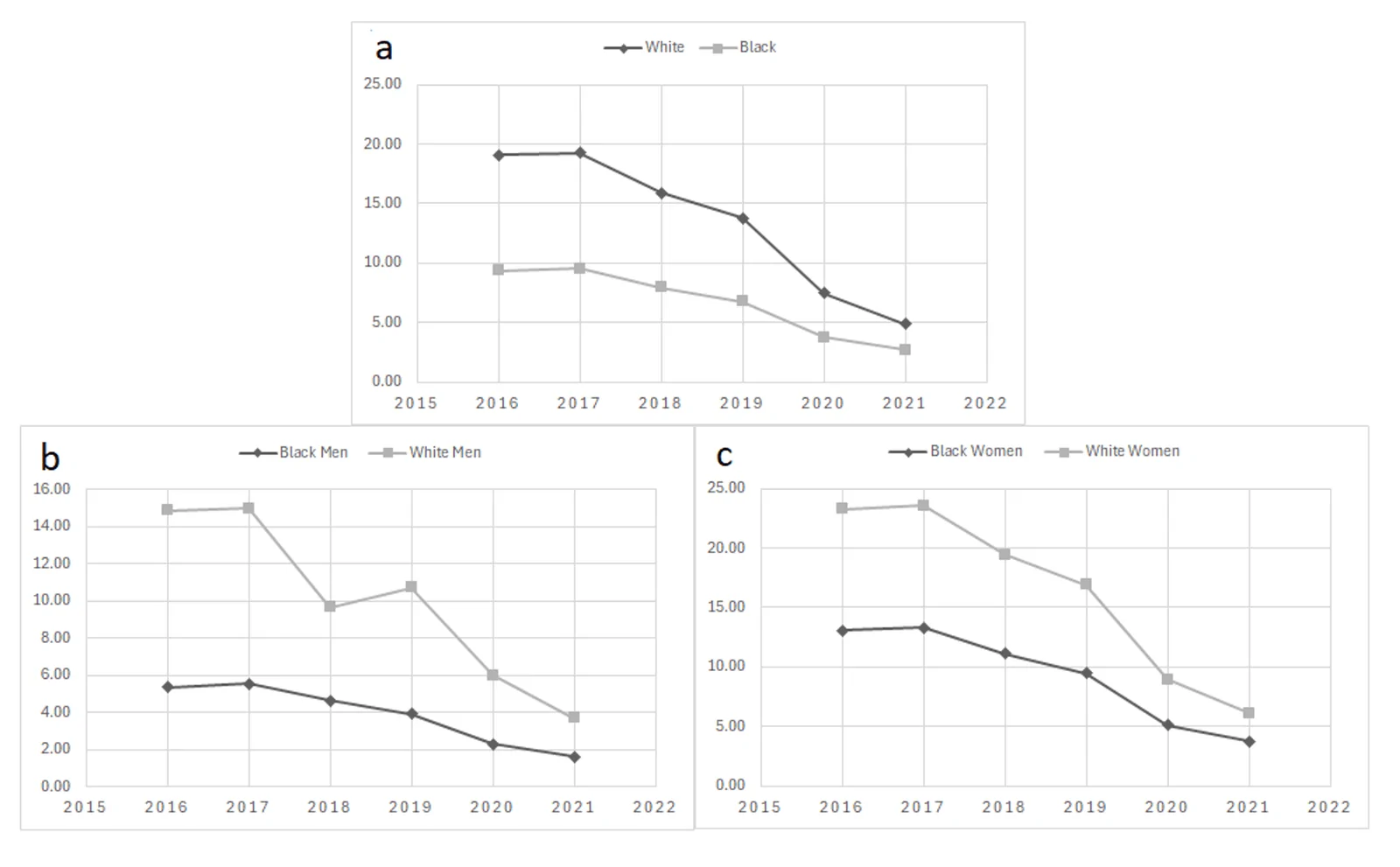
Age-adjusted TKA utilization disparities per 10,000 people by race and sex from 2016 to 2021. Panel (a): Trends for all White versus Black patients. Disparity slope = -1.0, 95% CI (-2.0, 0.1), p = 0.1. Panel (b): Trends for all White men versus Black men. Disparity slope = -1.3, 95% CI (-4.7, 2.2), p = 0.3. Panel (c): Trends for all White women versus Black women. Disparity slope = -1.0, 95% CI (-2.2, 0.1), p = 0.1. TKA: Total Knee Arthroplasty

Age-adjusted TKA utilization rates were 103.7% higher in White patients than in Black patients in 2016 (19.1 per 10,000 White patients, 9.4 per 10,000 Black patients). Rates decreased in both groups to 2021 (4.9 per 10,000 White patients, 2.7 per 10,000 Black patients), with no significant change in the White-Black disparity (p = 0.06) over the study period (Figure 3). In 2016, rates were 176.7% higher in White men than in Black men (14.9 per 10,000 White men, 5.4 per 10,000 Black men) and 77.5% higher in White women than in Black women (23.3 per 10,000 White women, 13.1 per 10,000 Black women). Rates decreased in all cohorts, with no significant change in the White men-Black men (p = 0.3) or White women-Black women (p = 0.06) disparities over the study period. Black men consistently had the lowest age-adjusted utilization rates, and White women consistently had the highest (Figure 3). 

## Discussion

Since 2010, policymakers and orthopedic societies have invested in reducing healthcare disparities. The DHHS instituted its first Action Plan to Reduce Racial and Ethnic Disparities in 2011. The plan aimed to reduce disparities in health insurance, quality of care, and provider diversity [9]. One of the four goals of the DHHS Healthy People 2020 initiative, released in 2010 for the decade, was to eliminate health disparities [9]. Despite these interventions, we found persistent White-Black disparities in TJA utilization rates and worsening disparities in THA utilization. This study incorporated HCUP-NIS datasets, which encompass 98% of the US population and 97% of all-payer hospital stays, making them well suited to analyze TJA utilization rates [18].

Multiple social science authors have called for an intersectional approach to quantitative research, emphasizing that race and sex are not mutually exclusive but instead interconnected, creating unique health disparities [13,19]. By stratifying our analysis by race and sex, our study offers a unique perspective on disparities in nationwide TJA utilization that may allow for more targeted public health interventions. Specifically, we found Black men to have the lowest TJA utilization rates. While the present study did not examine the etiology of these disparities, possible explanations include disparities that emerge earlier in the care pathway, including symptom recognition, referral patterns, and shared decision-making.

Prior studies have discovered similar White-Black disparities in TJA utilization rates [8]. A seminal study in the New England Journal of Medicine found worsening age-adjusted White-Black disparities in primary THA and TKA Medicare enrollee utilization rates from 1992 to 2001 [6]. Singh et al. found worsening age-adjusted White-Black disparities in primary TKA Medicare enrollee utilization rates but unchanged disparities for primary THAs from 1991 to 2008 [7]. Our study uniquely utilizes an all-payer dataset to provide an updated analysis of these disparities. This is important, as we found Black TJA patients were less likely than White patients to use Medicare. Only two prior studies have utilized HCUP datasets. Zhang et al. studied only TKA White-Black utilization rate disparities in 2001-2008 HCUP datasets, restricted to eight states, and found worsening trends [20]. Amen et al. utilized 2006-2015 HCUP-NIS datasets to find persistent, unchanged age- and sex-adjusted White-Black disparities in TKA and THA utilization rates [5]. Our study extends this analysis to 2021, utilizing the available HCUP-NIS datasets since the incorporation of the ICD-10 coding system. Disparities have since persisted, with THA utilization disparities significantly worsening over time.

The reason for White-Black disparities in TJA utilization rates is likely multifactorial. A qualitative study found that Black patients experience primary osteoarthritis pain as something they must just cope with [21]. In addition, Black patients with knee osteoarthritis are less willing to pursue TKA, with willingness associated with a better understanding of the procedure, more favorable expectations regarding LOS, outcomes, and postoperative pain, and greater trust in physicians [22]. Several other studies have observed differing utilization of healthcare interventions by Black compared with White patients. Black patients presenting to outpatient clinics were less likely to receive a knee MRI or knee intra-articular injections [23]. Within the orthopedic clinic, Black patients presenting with primary hip or knee osteoarthritis were less likely than White patients to receive TJAs [24]. In another study, they were less likely to receive a recommendation for TJA regardless of age or severity of osteoarthritis [25]. Furthermore, physicians' own implicit racial biases may also play a role [26]. While the present study found disparities in utilization rates, the underlying factors contributing to these disparities were not investigated and warrant future study.

Orthopedics continues to be primarily represented by White male physicians [27]. Hausmann et al. found that Black patients presenting to an orthopedic clinic with osteoarthritis were more likely to report prior healthcare experiences of racism, which negatively affected post-visit ratings of provider warmth and ease of communication [28]. Future studies should focus on primary care interventions, including patient outreach and education, clinician bias training, and equitable referral pathways, to address racial disparities in TJA utilization.

This study analyzes age-adjusted national TJA utilization trends stratified by sex and race at the national level. We found that White women consistently have the highest TJA utilization rates across all years. The White women-Black women disparity in THA utilization significantly worsened. Our findings conflict with population-based studies in Canada and Europe [11,12]. Hawker et al. found that, although women in Canada had a higher prevalence of hip or knee arthritis and worse disease severity, they were less likely than men to undergo TJA [11]. Jüni et al. found that women in Europe with hip pain were less likely than men to be referred to a specialist or to be on a waiting list for THA after adjusting for age and disease severity [12]. Differences in healthcare systems and population characteristics between the United States and Canada or Europe may explain these findings.

Racial disparities within the subset of women receiving TJAs have previously been studied. Cavanaugh et al. followed over 100,000 women enrolled in the 1993-1998 Women’s Health Initiative Clinical Trial and Observational Study, observing higher TKA utilization rates in White women than in Black women [29]. However, prior HCUP studies have noted that Black TKA patients are significantly more likely to be women [5,20]. This finding was also supported by our univariate analysis. Our population-based age-adjusted utilization rates show persistent White women-Black women TJA disparities, favoring White women. Black men also consistently had the lowest TJA utilization rates. To improve access to TJA care, future public health interventions should be specifically designed to address barriers faced by Black men with primary osteoarthritis. This may be further informed by qualitative studies aimed at understanding the persistent barriers in this population.

Several limitations to our study design are important to consider. As with all database studies, we cannot ensure accuracy in ICD-10 coding and variable entry into the dataset. However, the HCUP-NIS datasets remain nationally inclusive and well suited to studying TJA utilization trends [18]. Our utilization rates are not adjusted for existing diagnoses of osteoarthritis or disease severity. While prior studies have found Black patients to have increased rates of osteoarthritis compared with White patients, this does not reflect current national trends in the prevalence of the disease [3,4]. Our findings should be interpreted only in the context of the US Census age-stratified populations. Similarly, we do not consider racial and sex disparities in responses to conservative management of osteoarthritis that obviates the need for TJA. Future cross-sectional survey studies should be conducted to investigate racial and sex disparities in osteoarthritis symptoms and rates of conservative management. Additionally, our study is limited to an analysis of hospital admissions for TJAs, excluding ambulatory surgery center (ASC) procedures. Outpatient migration of THA/TKA may differ by insurance type, hospital status, socioeconomic status, or race, which may diminish or expand the disparities observed in the present study. However, a negligible proportion of elective TJAs in national Medicare claims data (2017-2019) were performed in ASCs. In 2020, ASCs accounted for 0% of THAs and 5% of Medicare TKAs, although same-day discharge following TJA increased during this period [30]. Same-day hospital TJA discharges were included in the analysis as patients with a LOS of zero days (n = 90,735), further supporting the accuracy of our findings. Another limitation is that the etiology of disparities in utilization rates was not investigated in this study. Utilization may differ due to disease burden, patient preference, referral patterns, insurance barriers, geographic access, and comorbidities, which future studies should account for. Finally, non-White and non-Black patients were excluded from analysis, which may limit the generalizability of our findings. Prior studies on utilization trends have also focused on White-Black disparities, and the results of the present study should be interpreted in that context. 

## Conclusions

In conclusion, national White-Black disparities in THA utilization worsened over time, while disparities in TKA utilization persisted without significant change. White women consistently had the highest utilization rates, and Black men consistently had the lowest. Future studies should investigate barriers to TJA care for Black men. At the same time, public health policies should implement multifaceted, equity-focused interventions, particularly for hip osteoarthritis, to reduce persistent TJA disparities. 
